# Shallot Species and Subtypes Discrimination Based on Morphology Descriptors

**DOI:** 10.3390/plants10010060

**Published:** 2020-12-29

**Authors:** Josipa Perković, Nikola Major, Dean Ban, Danko Cvitan, Smiljana Goreta Ban

**Affiliations:** 1 Institute of Agriculture and Tourism, Karla Huguesa 8, 52440 Poreč, Croatia; josipa@iptpo.hr (J.P.); nikola@iptpo.hr (N.M.); dean@iptpo.hr (D.B.); danko@iptpo.hr (D.C.); 2 Centre of Excellence for Biodiversity and Molecular Plant Breeding, Svetošimunska Cesta 25, 10000 Zagreb, Croatia

**Keywords:** *Allium* × *cornutum*, *Allium cepa* Aggregatum group, *Allium × proliferum*, PCA analyses, PLS

## Abstract

Shallots are an edible *Alliaceous* crop representing a group of genetically and morphologically different species. Shallot species determination is rather complex due to the high variability in phenotypes within a single species. Flower morphology has been successfully employed in shallot species determination; however, shallot florogenesis depends upon many genetic and environmental factors. There is a need for more accessible morphological descriptors used in shallot species determination, since flowering in shallot may not be consistent. In this study, we investigated the discriminating power of shallot vegetative and bulb morphology descriptors. European Cooperative Programme for Plant Genetic Resources morphology descriptors were used for describing 35 Croatian shallot accessions. The proposed methodology based on vegetative and bulb morphological descriptors could be used for shallot species discrimination. Additionally, two subtypes of *A. cepa* Aggregatum group were identified in this study: the first being the shallot type (1) and a potato onion type (2), which differed based on bulb morphology descriptors (bulb shape, bulb skin color, and a number of bulblets).

## 1. Introduction

Edible *Allium* species are one of the most important and represented vegetable crops in the world [1] as they refer to a number of species such as onion, garlic, leek, bunching onion, and different varieties of shallots [2,3]. According to Food and Agriculture Organization Corporate Statistical Database [1], onions are the third vegetable species produced in the world, after legumes and potatoes.

Shallot is a common name for genetically and morphologically different species and genotypes that are cultivated all over the world and characterized by the edible, vegetatively reproduced bulbs gathered in clusters [4,5,6]. The cultivation of shallot in Europe is based on either a small number of listed commercial cultivars, mostly from France and Italy (such are “Mikor”, “Jermor”, “French Grey Shallot”, “Giselle”, “Giselle Scalogno di Romagna”) or on the preservation of old landraces, ecotypes, and spontaneous hybrids in collections or home gardens [7,8,9,10].

Ex situ collections are a valuable source of plant material for further breeding programs, morphological characterizations of germplasm, assessing genetic erosion, and conservation of biodiversity [11,12]. Vegetatively propagated European *Alliums* including shallot landraces and cultivars are preserved in ex situ and core collections at the INRA, Ploudaniel, France; IPK, Gatersleben Germany; RIVGB, Olomouc, Czech Republic; RICP, Prague Czech Republic; NordGen and Swedish National Program for Diversity of Cultivated Plants [13]. At the same time, there is a vast number of morphologically diverse shallot cultivars in local and regional collections.

A high variability is present among shallot landraces from one group/genotype and obvious differences in phenotypes can be observed despite the landraces’ high kinship [14]. Rungis et al. [15] report on the genetic characterization of 264 potato onion accessions from Norway, Sweden, Finland, Lithuania, Latvia, Estonia, Czech, and Croatia gene bank collections. Scandinavian and Baltic countries were overlapping in clusters whereas Croatian accessions are clearly differentiated from all other countries [15]. The most cultivated European shallot species, the *A. cepa* Aggregatum group, is reported to have two bulb-forming subtypes [6,16]. The first type has narrow, pear-shaped bulbs, distinguishable from the second type, which is known as potato onion or multiplier onion with rounder bulbs and with many intermediate forms [6]. Fritsch and Friesen [2] are presenting different botanical classification of worldwide cultivated *A. cepa* Aggregatum group crops from the literature: *A. ascalonicum* auct. hort.; *A. cepa* var. *aggregatum* G. Don; var. *ascalonicum* Backer; and ssp. *orientalis* Kazakova, and state associated English names: shallot, potato onion, and multiplier onion. The two types of Aggregatum group shallots are often referred to in the literature as shallot and multiplier or potato onion [2,3,17]. The differentiation of the two types is based on the above-mentioned morphological differences in bulb size, shape, and number of clusters, but Fritsch and Friesen [2] emphasize how they are not easily distinguishable, and many intermediate forms could be found in this group. The Swedish standard for nomenclature of cultivated plants differentiates between potato onion and shallot but states how borders are unclear [18]. Therefore, a systematic approach to morphological, genetic, and biochemical characterization of shallot cultivars is of utmost importance to identify genotypes in collections and to unravel relations between them, especially regarding the differentiation between shallots and potato onions both form the *A. cepa* Aggregatum group.

As a first tool in the process of describing and mapping plant collections, plant gene banks use vegetative, agronomic, and generative morphology descriptors and passport data such as those of the European Cooperative Programme for Plant Genetic Resources (ECPGR) [19]. Morphology descriptors databases of ex situ edible plant collections are essential for further research, ethnobotanical studies, and breeding programs aimed toward agronomic, nutritive, and bioactive favorable traits as well as giving context to genetic research.

The available literature suggests that all collected shallot landraces in Croatia belong into three species [5]. Shallots from the *A. cepa* Aggregatum group (2*n* = 2x = 16), belong to the *A. cepa* species. The second species, *A. × proliferum* (Moench) Schrad (2*n* = 2x = 16), is a hybrid of *A. cepa* and *A. fistulosum*. The third shallot species, *A. × cornutum* Clementi ex Vis. (2*n* = 3x = 24), is of a unique triparental origin: *A. cepa*, *A. pskemense,* and *A. roylei* [5,6,20]. Landraces of *A. × cornutum* are grown mainly in the Mediterranean part of Croatia [5], whereas landraces from the *A. cepa* Aggregatum group are mostly distributed in the mainland.

Considering the rising number of shallot accessions in ex situ collections, there is an increasing need for fast and simple tools for species determination. Studies by Puizina [5] and our earlier study by Major et al. [21] showed the significance of flower morphology in shallot species determination and a good correlation with the results of genetic research and bioactive compounds and minerals. However, flower morphology is not always available nor a simple tool for shallot determination, since not all accessions or genotypes will or can flower in the field. Florogenesis in *Alliums* is a complex process that is dependent on many genetic and environmental factors [2,22]. Therefore, more accessible morphological traits that could be employed as diagnostic tools should be determined. Relevant diagnostic characteristics of *Alliums*, among others, imply bulb morphology, leaf number, leaf shape and size, and geographical distribution data [23].

The earlier study of Major et al. [21] showed how multivariate analyses based on vegetative and bulb morphological traits of onions and other *Alliums* could be used to discriminate among species. This study continues our earlier work and findings aiming to simplify the determination process of shallot landraces and the complexity of their diversity. Multivariate analyses were used to differentiate onion cultivars [11,24,25], garlic cultivars [26], and shallot accessions [14,27,28]. Partial Least Squares (PLS) regression is a type of a supervised multivariate analysis that forms new components that explain most of the variance of the independent variables and is useful for predicting dependent variables reducing the dimensionality of the dataset [29]. PLS is often used when there are many independent variables and comparatively little data [29,30]. In this study, Partial Least Squares Discriminant Analysis (PLS-DA) modeling was applied as an exploratory tool for the identification of vegetative and bulb morphology descriptors critical in the discrimination between shallot species.

As already mentioned above, the phenotype diversity of shallot’s local landraces places difficulties in shallot species determination. Unclear differences between shallot species or subtypes are mainly a result of either inconsistent plant material or a too restricted list of morphological descriptors [14]. This research aims to determine if discrimination between shallots species could be achieved using vegetative and bulb morphology, primarily ECP/GR descriptors. In addition, several new morphological descriptors will be introduced as key features for shallot species differentiation, as more accessible traits compared to flower morphology. By increasing the descriptor list and introducing bulb morphology, we are aiming toward a simplified model for accession discrimination and to contribute to the complex debate on *A. cepa* Aggregatum group subdivision on shallot and potato onions.

## 2. Results

### 2.1. Comprehensive Morphological Data of the Ex Situ Collection of Croatian Shallot Landraces

#### 2.1.1. Flower and Inflorescence Morphology

Based on the number of weeks after planting (WAP) acquired until flowering, the Croatian accessions were divided into three groups: 31 WAP, 32 WAP, and 33 WAP (Table 1 and Table S1). Croatian accessions were also divided based on the percentage of plants that flowered: no or very rare flowering, rare, most to obligatory flowering (Table 1 and Table S1).

Most of the *A. cepa* Aggregatum group accessions had starlike perianth type with a green stripe (FQL 5 {1}), unlike campanulate, green striped perianth {2} for *A. × proliferum*, or a starlike perianth with a purple stripe {3} for *A. × cornutum* (Table 2). *A. cepa* Aggregatum accessions recorded also round inflorescence with no bulbils (FQL 6 {1}), which was different from inflorescence with bulbils {3} and {2} as seen in *A. × cornutum* and *A. × proliferum* accessions. The pistils having a lower position than the stamens (FQL 4 {1}) differentiates *A. cepa* Aggregatum group accessions from others (Table 2). According to Table 2, *A. × cornutum* accessions have yellow anthers and *A. cepa* stamen morphology (FQL 3 {3}), in contrast to green anthers and either simple {2} or *A. cepa* stamen morphology {1} as found in *A. cepa* Aggregatum group and *A. × proliferum* accessions.

*A. × proliferum* accessions have a scape type that carries bulbils in several levels (FQL 7 {2}), including the widest scape in diameter (FQN 6) and inflorescence with a smaller flower number (FQN 1) compared to other species accessions (Table 2).

The widest inflorescence (FQN 1) is found in the *A*. *cepa* Aggregatum group (42.83 ± 1.02 mm) and those accessions differed from *A*. × *proliferum* (30.69 ± 3.67 mm), whereas the inflorescence observed in *A*. *× cornutum* was in between (38.64 ± 1.08 mm), as seen in Table 3. Statistically, there is no difference between the stamen length of shallot species, and *A. × proliferum* accessions could be seen between the *A. cepa* Aggregatum group and *A. × cornutum* in length. *A. cepa* Aggregatum group accessions are different from other two species by petal length (FQN 4), as these accessions measured the shortest petal lengths (3.58 ± 0.04 mm), as seen in Table 3. The ANOVA of quantitative flower variables showed that the *A. cepa* Aggregatum group accessions have the smallest petal diameter (FQN 5) of only 2.13 ± 0.03 mm (Table 3), which differentiates them from the other two species. The widest scape diameter (FQN 6) was recorded in *A. × proliferum* accessions, 28.36 ± 0.94 mm compared to 14.49 ± 0.23 mm in the *A. cepa* Aggregatum group and 9.94 ± 0.44 mm in *A. × cornutum* accessions (Table 3).

**Figure 1:**
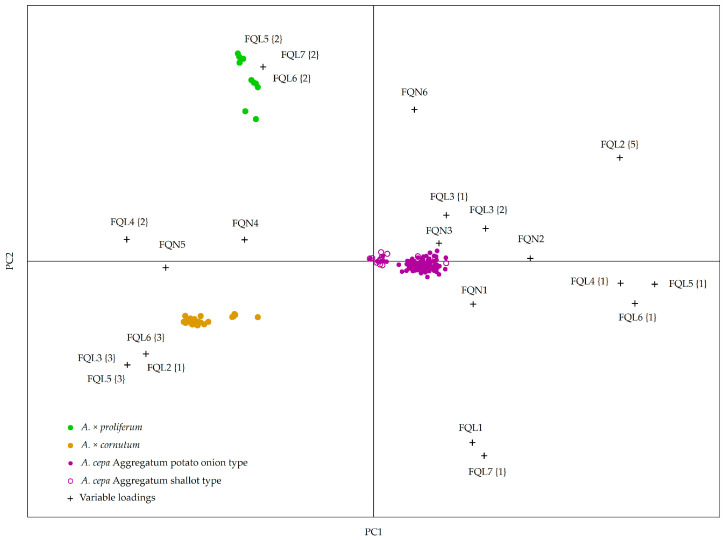
Principal Components Analysis (PCA) biplot based on generative descriptors for 32 flowering shallot accessions from Croatia. Five individual plants per accessions were measured; each dot in the biplot represents one individual measurement.

Based on the PCA of 13 qualitative and quantitative flower morphology descriptors, FQL and FQN respectively (Table 2 and Table S2), accessions were divided into three distinct groups, as seen in Figure 1.

The first four main components, with eigenvalues greater than 1, described 83.9% of the total variance (Table S3). The first principle component (PC1) represents 40.4% of the total variance (Table S3) and is correlated with variables regarding the anther color (FQL 2), stamen type (FQL 3), pistil type (FQL 4), perianth type (FQL 5), inflorescence type (FQL 6), and petal diameter (FQN 5) (Figure 1). Variables having the highest loading scores with PC1 and separating accessions of the *A. cepa* Aggregatum group from the accession of two other shallot species are perianth (FQL 5 {1}), inflorescence (FQL 6 {1}), and pistil types (FQL 4 {1}) and petal diameter (FQN 5), as seen in Figure 1 and Table S4. Descriptors for anther color (FQL 2 {1}) and stamen type (FQL 3 {3}) are correlating with PC1, and they separate *A. × cornutum* from other two species (Figure 1).

Descriptors having the highest eigenvector value and correlating with the second principal component, PC2 (26.8% of total variance), are the scape type (FQL 7), scape diameter (FQN 6), and inflorescence flower number (FQL 1), which separate *A. × proliferum* accessions from the two other species (Figure 1, Table S4).

The highest contribution on PC3 (11.2% of total variance) have flower pedicle (FQN 2) and inflorescence diameter (FQN 1), as seen in Table S3.

The fourth main component described 5.5% of the total variance and correlates with variables describing the stamen and petal length (FQN 3 and FQN 4) and stamen type (FQL 3 {3}) separating *A. × cornutum* from *A. cepa* Aggregatum group accessions, whereas *A. × proliferum* accessions are in the middle (Table S3, Figure 1).

#### 2.1.2. Vegetative and Bulb Morphological Traits

Great diversity was observed among shallot species and accessions regarding qualitative vegetative descriptors used in this study (Table S4). Accessions of the *A. cepa* Aggregatum group have the highest diversity regarding leaf color, foliage cracking, and the degree of leaf waxiness with either green (VQL 1 {3}), bluish green (VQL 1 {6}), gray-green (VQL 1 {4}), or even dark green (VQL 1 {5}) leaf color and strong (VQL 3 {7}), medium (VQL 3 {5}), or weak (VQL 3 {3}) foliage cracking as well as weak (VQL 4 {3}) to strong (VQL 4 {7}) degree of leaf waxiness, as seen in Table S4. Accessions belonging to *A. × proliferum* are consistent in dark green leaves (VQL 1 {5}), erect leaf attitude (VQL 2 {7}), and a strong degree of leaf waxiness (VQL 4 {7}), while the other species have more diverse traits for these variables (Table S4). According to the data presented in Table S4, *A. × cornutum* accessions have weak foliage cracking (VQL 3 {3}) and light green or green foliage color (VQL 1 {1} or {3}).

**Figure 2:**
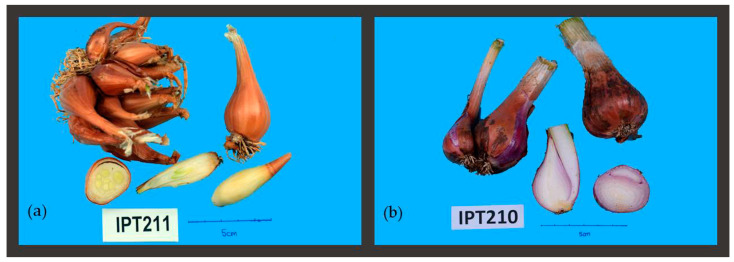
Shallot species: *A. × cornutum* (**a**) and *A. × proliferum* (**b**) cluster and bulb images. The number in the figure represents the accession code assigned in the ex situ collection at IPTPO.

According to the data in Table 4, accessions of *A. × cornutum* differ from other two species in ovate bulb shape (BQL 1 {7}), having no bulblets offsets (BQL 3 {0}), light brown bulb skin color (BQL 4 {4}), thick bulb skin (BQL 5 {7}), green/white bulb flesh color (BQL 6 {3}), as seen in Figure 2a. Despite having many bulbs in one cluster, they are separated in their tunic and have no offset underground bulbs, as seen in equatorial and longitudinal bulb dissection (Figure 2a). *A. × proliferum* accessions also consistently have two bulbs in cluster (BQN 1). They have the widest cluster diameter (BQN 3) and bulb shape, which is either ovate or broad oval (Table 4; Figure 2b).

**Figure 3:**
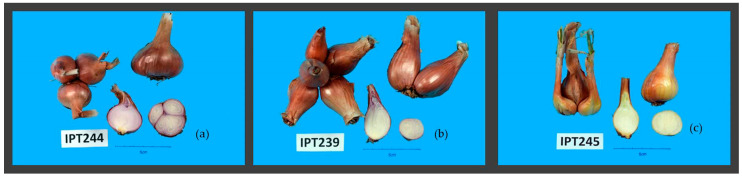
Diversity among *A. cepa* Aggregatum group species accessions bulb images. Broad oval bulb shape in potato onion type (**a**); ovate, pear-shaped bulbs in shallot type (**b**,**c**); white bulb flesh and yellow tunic color (**c**). The number in the figure represents the accession code assigned in the ex situ collection at IPTPO.

Accessions of *A. cepa* Aggregatum group species are the most diverse having different bulb shapes. Mostly, *A. cepa* Aggregatum group accessions have a broad oval shape of mature dry bulbs (Table 4, Figure 3a), but in accessions IPT239, IPT240, IPT227, and IPT245, we recorded an oval bulb shape (Table 4, Figure 3b,c), as seen in *A. × cornutum* (Figure 2a). *A. cepa* Aggregatum group accessions mostly have violet/white bulb flesh color except for IPT227, IPT241, and IPT245 (Table 4, Figure 3c). The majority of the *A. cepa* Aggregatum group accessions will have bulblets present as offsets either inside the thin outer bulb tunic (Figure 3a) or as a small side bulb, but in the case of accessions IPT208, IPT230, IPT239, and IPT240, they were absent (Table 4, Figure 3b,c). Most of the *A. cepa* Aggregatum group accessions have a light violet bulb skin color except for accessions IPT227, IPT40, IPT241, IPT242, IPT243, IPT244, and IPT245 (Table 4).

According to the vegetative and bulb morphology data presented in Table 4 and Table S4, two subtypes emerged from the *A. cepa* Aggregatum group corresponding to the potato onion type and shallot type suggested by Leino et al. [14]. Accessions belonging to the *A. cepa* Aggregatum subtype shallot type (IPT227, IPT239, IPT240, IPT241, and IPT245) differ from the *A. cepa* Aggregatum potato onion type in ovate bulb shape (BQL 1), yellow bulb skin color (BQL 4), and a smaller number of bulblets (BQL 2 and BQN 1) compared to the broad oval shape and light violet skin color of bulbs of the potato onion type (Table 4).

ANOVA of quantitative vegetative and bulb descriptors based on four groups (three proposed species and two subtypes) shows statistically significant differences between *A. cepa* Aggregatum group subtypes. The *A. cepa* Aggregatum potato onion type has a wider leaf diameter (10.66 ± 0.17 mm) than the *A. cepa* Aggregatum shallot type (9.24 ± 0.35 mm). The *A. cepa* Aggregatum potato onion type also has the highest number of bulbs in the cluster (12.10 ± 0.34 no./cluster) and higher cluster weight (273.12 ± 5.94 g/cluster) compared to the *A. cepa* Aggregatum shallot type clusters (7.67 ± 0.69 no./cluster), 171.62 ± 12.17 g/cluster, respectively), as seen in Table 5. In Table 5, it is shown how *A. × proliferum* accessions have the widest leaf diameter (18.71 ± 0.64 mm) but the lowest number of bulbs in cluster (2.01 ± 1.26 no./cluster) compared to other shallot species (Table 5).

### 2.2. Creating a Model for Accession Grouping Based on Bulb and Vegetative Organ Morphological Descriptors

**Figure 4:**
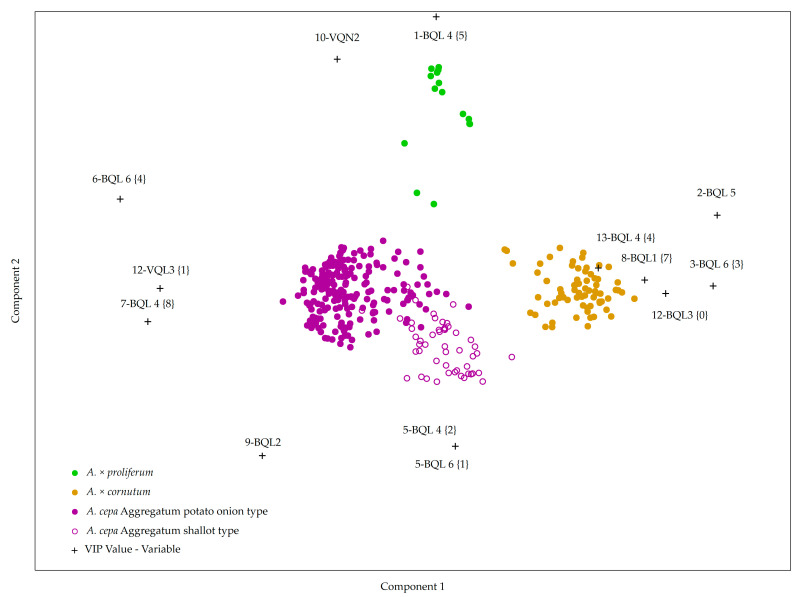
Partial Least Squares Discriminant Analysis (PLS-DA) biplot of comprehensive ex situ collection of Croatian accessions based on qualitative and quantitative vegetative and bulb morphology descriptors (VQL, VQN, BQL, and BQN). Ten individual plants per accessions were measured; each dot in the biplot represents one individual measurement.

Partial Least Squares Discriminant Analysis (PLS-DA) of vegetative and bulb descriptors was employed to determine if these morphological traits could be used for shallot species prediction as an alternative to the established flower morphology descriptors. Based on vegetative and bulb morphology, the PLS-DA biplot shows the differentiation of shallot accessions into four groups (Figure 4). There are overlaps in groups, mainly between accessions belonging to the *A. cepa* Aggregatum potato onion type and to the *A. cepa* Aggregatum shallot type (Figure 4). Accessions belonging to the same species are mainly grouped together. In addition, differentiation among most accessions in the *A. cepa* Aggregatum group, which is based on all vegetative and bulb morphological characteristics presented in this study, is consistent with the potato onion and shallot subtype grouping.

Vegetative and bulb traits that separate the *A. cepa* Aggregatum potato onion subtype from the *A. cepa* Aggregatum shallot subtype as well as the *A. × cornutum* species are ovate bulb shape (BQL 1 {7}), absence of bulblets (BQL 3 {0}), and yellow bulb outer skin color (BQL 4 {2}), as seen in Figure 4.

Descriptors differentiating between the *A. cepa* Aggregatum group and the *A. × cornutum* are bulb skin thickness (BQL 5) and bulb flesh color (BQL 6 {4} or {1}), as *A. × cornutum* accessions have thick bulb skin (BQL 5) and green/white flesh color (BQL 6 {3}) compared to *A. cepa* Aggregatum group accessions with thin bulb skin (BQL 5) and violet-white (BQL 6 {4}) or white (BQL 6 {1}) flesh color (Table 4, Figure 3). Brown bulb outer skin color (BQL 4 {5}) and the widest leaf diameter (VQN 2) are descriptors that have the highest discriminating power separating the *A. × proliferum* from other two species (Figure 4). Additional descriptors separating *A. × proliferum* from the other species are leaf waxiness (VQL 4), foliage attitude (VQL 2), and number of bulbs per cluster (BQL 2), as these accessions possess strong leaf waxiness, erect attitude in leaves, and a scarce number of bulbs per cluster (Table S4).

## 3. Discussion

In this study, we used 28 morphological qualitative and quantitative descriptors to describe and group 35 Croatian shallot accessions according to species. As Puizina [5] suggested and we confirmed in our earlier study of 11 flowering Croatian shallot accessions in Major et al. [21]: flower morphology descriptors can be used for the accurate determination of shallot species. This study confirmed the proposed methodology and results based on the PCA and ANOVA of flower morphology descriptors presented in Major et al. [21].

Accessions of the *A. cepa* Aggregatum group, both types, potato onion and shallot type, differed from *A.* × *cornutum* and *A.* × *proliferum* based on starlike perianth with a green stripe (FQL 5 {1}), round inflorescence with no bulbils (FQL 6 {1}), pistil lower than stamens (FQL 4 {1}), smallest petal diameter (FQN 5), longest pedicle (FQN 2), and shortest petal length (FQN 4). Accessions belonging to the *A. × cornutum* species could be distinguished from the *A. × proliferum* and *A. cepa* Aggregatum group by possessing a combination of yellow anthers and *A. cepa* stamen morphology (FQL 2 {1}, FQL 3 {3} respectively) not present in the accessions of the other two species. Accessions of the *A. × proliferum* species were separated from the *A. × cornutum* and *A. cepa* Aggregatum group based on the widest scape carrying bulbils in several levels (FQN 6 {2}, FQL 7 {2} respectively) and inflorescence smaller in size with the lowest flower number (FQN 1). Flower number in umbel (FQL 1) proposed in Major et al. [21] was shown not to be reliable in differentiating *A. × cornutum* from the *A. cepa* Aggregatum group. According to PCA and ANOVA, we divided 32 shallot accessions into three species based on their flower morphology: two as *A. × proliferum* (IPT023, IPT210), five as *A. × cornutum* (IPT021, IPT211, IPT212, IPT213, and IPT214), and 25 as *A. cepa* Aggregatum group: accessions IPT176, IPT208, IPT217, IPT218, IPT225, IPT226, IPT228, IPT229, IPT230, IPT231, IPT232, IPT233, IPT234, IPT235, IPT236, IPT237, IPT238, IPT242, IPT243, and IPT244 in *A. cepa* Aggregatum potato onion type and accessions IPT227, IPT239, IPT240, IPT241, and IPT245 in *A. cepa* Aggregatum shallot type.

In our collection, the variability in number of days from planting to flowering was observed for shallot plants grown under the same environmental conditions. Accessions from the *A. cepa* Aggregatum group and *A. × proliferum* species flowered 31 or 32 WAP; however, accessions belonging to the *A. × cornutum* species flowered later than that, at 33 WAP. In addition, the number of flowered plants ranged from around 0% for a few accessions (IPT022, IPT215, and IPT216) to more than 70% of flowered plants in the majority of accessions. The lack of uniform flowering can be attributed to the dependence of florogenesis on both genetic and environmental factors [2,22,31,32]. The flowering function in shallots of the *A. cepa* Aggregatum group has been associated with shLFY genes based on which shallot genotypes occur as either a naturally flowering type (abundant, obligate flowering), inducible flowering type (by favorable environmental factors) or non-flowering genotypes [33]. It was observed that colder temperatures during bulb storage and in the early growth period result in faster shallot bolting and flowering; however, temperatures exceeding 30 °C in storage or in the field can reduce the rate and percentage of plants entering the generative phase [10,31]. When the genetic background is accompanied with environmental factors favorable for flowering (exposure to temperature of 5 to 10 °C) and certain bulb physiological age, the transition from vegetative to the generative developmental stage could be triggered [34,35]. In our previous study of Major et al. [21], we also observed inconsistencies in the flowering of several shallot accessions, as they produced either no inflorescence or just a small subset of plants flowered [21]. In the following year, we updated the collection by adding 22 new accessions, and again, we encountered the absence of flowering in accessions IPT022, IPT215, and IPT216; therefore, employing the inflorescence morphology descriptor for the species annotation of all accessions was rendered impossible.

Variations of the leaf’s qualitative characteristics, namely leaf color, foliage growth, and foliage cranking were already considered as a possible criteria to differentiate *A. cepa* varieties by Ahmed et al. [36] and in *Allium* species from Iran by Aryakia et al. [27]. Laila et al. [37] reported number of leaves as a characteristic differentiating 16 shallot cultivars from Indonesia. Morphological variations on *Allium* spp. based on leaf quantitative characteristics, including leaf length, leaf width, and number of leaves per individual plant were observed in the study by Fitriana and Susandarini [28] and Khosa et al. [11].

In this study, most vegetative traits differing species one from another are in accordance with findings in Major et al. [21]. *A. × cornutum* accessions are distinctive in their narrowest leaf diameter (VQN 2), light green or green foliage color (VQL 1 {1} or {3}), and weak foliage cracking (VQL 3 {3}). Accessions of *A. × proliferum* could be differentiated based on their vegetative gigantism: dark green leaves (VQL 1 {5}), erect leaf attitude (VQL 2 {7}), strong degree of leaf waxiness (VQL 4 {7}), and widest leaf in diameter (VQN 2). Puizina [5] also reports on vegetative gigantism in *A. × proliferum* and *A. fistulosum*, which is a species she reports as parental to the hybrid *A. × proliferum* [5,38]. *A. × proliferum* is a hybrid species, which propagates vegetatively from inflorescence bulbils or top sets, formerly known as *Allium cepa* var. *viviparum* (Metzger) Alefeld, usually named as Egyptian onion, tree, or top onion and in Croatia named ‘Ljutika-talijanka’ [38]. For *A. × proliferum*, the author reports either mixed inflorescence, containing both bulbils and sterile flowers, or having only bulbils [5,38]. Puizina [5] also observed how bulbils of *A. × proliferum* often sprout within the transformed umbel, sometimes in two or more levels, as we recorded in this study for accessions IPT023 and IPT210.

Schwinn et al. [39] and Fitriana and Susandarini [28] marked the role of bulb color as an important discrimination trait in *A. cepa* and shallot cultivars. Khandagale and Gawande [40] underlined the importance of bulb color as a quality indicator in the *A. cepa* breeding programs and a criterion for variety classification. According to literature suggestions, some bulb descriptors (ECPGR) were recorded in these studies that have not been featured in Major et al. [21]. Those are bulb skin color (BQL 4), bulb skin thickness (BQL 5), and bulb flesh color (BQL 6). The descriptors are added in order to improve accession separation and to find the appropriate morphology species indicator.

According to the PLS-DA model based on vegetative and bulb descriptors, accessions of *A. × proliferum* and *A. × cornutum* are grouped separately and in accordance with the results obtained by flower morphology descriptors. *A. × proliferum* accessions can be separated from the other two species by leaf diameter (VQN 2). *A. × proliferum* accessions have on average two bulbs per cluster, the widest cluster diameter (BQN 1, BQN 3), medium thick bulb skin, brown color (BQL 5 {5}, BQL 4 {5} respectively), and the widest leaf diameter (VQN 2). Puizina [5] already reports on gigantism regarding the vegetative growth of *A. × proliferum*. Major et al. [21] also identified the widest leaf diameter and the smallest number of bulbs in cluster for *A. × proliferum* compared to *A. × cornutum* and the *A. cepa* Aggregatum. In this study, *A. × cornutum* accessions are distinctive in several bulb traits, including ovate bulb shape (BQL 1 {7}), no bulblets offsets (BQL 3 {0}), thick bulb skin, and light brown color (BQL 5 {7}, BQL 4 {4}, respectively), green/white bulb flesh color (BQL 6 {3}) and have the smallest bulb diameter compared with the two other species. Fredotović et al. [20] and Puizina [5] reported on the elongated pear-shaped bulbs in *A. × cornutum,* and our earlier study by Major et al. [21] supports the finding that the *A. × cornutum* has the narrowest bulb diameter compared to other shallot species from Croatia, which contributes to the further identification of *A. × cornutum* and its specific features compared to other shallot species and types later assessed and later examined in this study.

A differentiation based on bulb morphology descriptors among *A. cepa* Aggregatum group accessions in the PLS-DA model can be observed, one group being the potato onion type and the other being a shallot type. The *A. cepa* Aggregatum shallot type is morphologically very close to the *A. × cornutum* accessions and is separated from the *A. cepa* Aggregatum potato onion type based on a combination of bulb morphology descriptors: its ovate bulb shape, small number of bulbils, and yellow bulb skin color. The entire *A. cepa* Aggregatum group could be distinguished from *A. × cornutum* and *A. × proliferum* by two descriptors: bulb skin thickness and bulb flesh color. Our findings are in accordance with reports on the two phenotypes of bulbs in the *A. cepa* Aggregatum group [6,14]. French shallot breeding programs also state how shallot cultivars (*A. cepa* Aggregatum) are grouped into long and short Jersey shallot describing either long or rounder bulb shape in red fleshed shallots [7,13]. Rabinowitch and Kamenetsky [6] report on the shallot type of bulb in the *A. cepa* Aggregatum group: a narrow, ovoid to pear-shaped bulbs with red-brown (coppery) tunic, which corresponds with the *A. cepa* Aggregatum shallot type isolated in this study from the *A. cepa* Aggregatum group. Brewster [17] also specifies how potato onions have rounder bulbs, wider rather than longer in shape, and have between three and 20 bulbs in a single cluster, which are gathered and encased by the dry, outer bulb skins differing from shallot type and its narrow, separate bulbs. The above-mentioned findings of Brewster [17] are in accordance with our study, and differences between potato onion type and shallot type of the *A. cepa* Aggregatum group are showing differences regarding the descriptors of bulb shape (BQL 1) and number of bulblets offsets (BQL 3). Bulb shape could be influenced by planting season: elongated shaped bulbs originated from the autumn planting and round bulbs originated from the spring planting season [41]. To mitigate the above-mentioned environmental impact on bulb shape, in our study, all of the accessions were planted at the same time in early autumn. The *A. cepa* Aggregatum group accessions showed high polymorphism in bulb morphology. They ranged from broad oval {4}, ovate {7}, globe {5}, or rhomboid {3} bulb shape (BQL 1) and had mostly violet/white {4} bulb flesh color, while several accessions were white {1} (BQL 6). Earlier reports of Rabinowitch and Kamenetsky [6] are confirmed in our study: round and broader oval bulbs of *A. cepa* Aggregatum accessions of potato onion type are distinguished by PLS-DA from ovate or pear-shaped bulbs of the *A. cepa* Aggregatum shallot type (IPT227, IPT239, IPT240, IPT241, and IPT245) as well as from the *A. × cornutum* species.

The bulb skin color (BQL 4) of the *A. cepa* Aggregatum group accessions ranged from light violet {8} to light brown {4} and yellow {2}. Dahlen et al. [42] and Arifin et al. [43] also report on shallot tunic color (dry skin surrounding bulbs) ranging from reddish-purple to brown or yellow. Accessions IPT227, IPT240, and IPT245 are yellow in bulb skin color and white in bulb flesh color unlike other *A. cepa* Aggregatum group accessions. This difference in color of bulbs was already noticed by Sulistyaningsih et al. [44], where they also reveal how rare white and common red colored bulbs of the Indonesian shallot do not differ in both chloroplast and mitochondria DNA, karyotype, anther morphology, or leaves. In the reported study, it is presumed how a white bulb shallot is a result of a natural mutation process of a more common red bulb shallot [44].

A higher cluster weight (BQN 2) and the number of bulbs per cluster (BQL 2 and BQN 1) are descriptors separating the *A. cepa* Aggregatum potato onion type from the *A. cepa* Aggregatum shallot type. A characteristic of the *A. cepa* Aggregatum potato onion type is to form many bulbs and bulblets per cluster as well as many daughter bulbs inside the same dry bulb tunic. This feature was already noticed and well documented by Krontal et al. [22]. The authors report on the Aggregatums’ ability to form lateral buds in the axils of the inner blade-bearing leaves, so they not only stay attached on the original basal plate after becoming side bulbs, but they remain in the original bulb tunic as one big bulb [22]. Brewster [17] also informs on *A. cepa* Aggregatum potato onions having between three and 20 bulbs gathered together in a cluster, which is encased by the dry, outer bulb skins. This trait was not detected in the *A. cepa* Aggregatum shallot type.

Even tough cluster bulb weight and the number of bulbs per cluster are influenced by environmental factors; all studied accessions were planted in the same trial field at the same time, with similar agricultural measures as in the previous study by Major et al. [21]. Nevertheless, the differences in bulb weight across harvest years have little impact on the obtained differentiation between shallot species.

The variability observed in the morphological traits of Croatian shallot landraces could be utilized in future breeding programs particularly aimed in selection of varieties adjusted to organic production and/or diverse climatic constraints.

## 4. Materials and Methods

As a part of the National Program for the Conservation and Sustainable Use of Plant Genetic Resources for Food and Agriculture, shallot landraces were continuously collected from 2014 to 2018 from different parts of Croatia (Table S1), and collected plant material was vegetatively propagated and preserved every year under the same field conditions of the ex situ experimental farm on the Institute of Agriculture and Tourism in Poreč, Croatia, IPTPO (N 45°13′20.30″, E 13°36′6.49″). In the year 2018, all 35 shallot accessions were planted in the experimental farm of the IPTPO. One bulb from a cluster was planted at a distance of 20 cm in a row and 30 cm between rows, with 40 bulbs in total planted for each accession. The bulbs of standard size for each accession were chosen for planting. Agricultural practices for onion growing without irrigation were applied, and the weeds were removed manually [4]. Fertilizer NPK (5:20:30) was incorporated at 500 kg ha^−1^ before planting, and 45 kg ha^−1^ N was applied (urea source) at the beginning of March. The flowering period was from 6 June to 20 June 2018. Shallots were harvested at the beginning of July, when 50% of pseudo stems were bent over and left to cure under shade for a month.

Morphological data were recorded in 2018 during the vegetation period (qualitative and quantitative vegetative descriptors, VQL and VQN), the flowering period (qualitative and quantitative flower and inflorescence descriptors, FQL and FQN) and during post-harvest and curation (qualitative and quantitative bulb descriptors, BQL and BQN). Descriptors for generative organs are in accordance with Puizina [5], Major et al. [21], and ECPGR descriptors for vegetatively propagated *Allium* species [19]. The ECPGR descriptors were also used for vegetative and bulb morphology description. A total of 17 qualitative and 11 quantitative ECPGR descriptors were used. A morphological description of flowering shallot accessions was conducted on 5 plants, while vegetative and bulb descriptors were recorded on 10 plants per accession. Additionally, bulbs from four commercial *Allium* cultivars were purchased on the local market to compare the antioxidant activity, phenolic, and dry matter content with the local shallot accessions.

### Statistical Analyses

Data on quantitative morphological descriptors were analyzed by analysis of variance (ANOVA). Principal Components Analysis (PCA) was performed on both qualitative and quantitative morphological descriptors of shallot flowering accessions using the Non-linear Iterative Partial Least Squares (NIPALS) algorithm [45]. Partial Least Squares Discriminant Analysis (PLS-DA) was performed on vegetative and bulb morphology descriptors of all shallot landrace accessions by also employing the NIPALS algorithm [46]. Prior to multivariate analyses, nominal variables were transformed using the one-hot-encoding method where a single variable with *n* cases and *z* observations was transformed to *z* binary variables with *n* cases [47]. All data were standardized with the Z-transform function [48]. Statistical analysis was performed using Statistica 13.4.0.14. Significant differences were determined at *p* ≤ 0.05 and homogenous group means were compared by Tukey–Kramer Unequal N HSD test.

## 5. Conclusions

The proposed methodology of discrimination based on vegetative and bulb properties could be used for distinguishing all three shallot species *A. × cornutum, A. × proliferum,* and *A. cepa* Aggregatum group observed in this study. Aside from flower morphology, descriptors with the most discriminating power are bulb skin color and thickness, bulb flesh color, bulb shape, leaf diameter, and number of bulbs in cluster, and all should be applied simultaneously for the best results.

The obtained results also indicate how two phenotypes or subtypes among the *A. cepa* Aggregatum group could be distinguished in the IPTPO ex situ collection. The first proposed subtype is the *A. cepa* Aggregatum shallot type, which is characterized by ovate, longer, or pear bulb shape and the absence of daughter bulblets in dry tunic, whereas the second is the *A. cepa* Aggregatum potato onion type, which is characterized by a round and flat bulb shape with many daughter bulbs inside the tunic.

In this study, for the first time, we described and performed analyses on the entire set of morphology descriptors, both ECPGR descriptors for shallot and onion as well as additional botanical descriptive traits, which, to the best knowledge of the authors, was not published previously. The literature available so far used only a few descriptors to describe the subtypes in the *A. cepa* Aggregatum group, whereas in the PLS-DA model of this study, many descriptors were evaluated simultaneously.

This study provides faster tools for discrimination between shallot species and subtypes for gene banks. The use of proposed morphological traits of the bulb and vegetative plant parts could be more feasible for small collections and gene banks where molecular studies are not possible or available. In addition, this study raises questions concerning further investigations on shallot kinship, especially in the field of molecular and biochemical characterization.

## Figures and Tables

**Table 1:** Flowering time and ability to flower of Croatian shallot accessions.

Flowering Time in 2018 (WAP) ^1^		Accessions ^2^
31 WAP		IPT176, from IPT226 to IPT229, from IPT237 to IPT245
32 WAP		*A. × proliferum* accessions, IPT208, IPT216, IPT217, IPT218, IPT225, from IPT230 to IPT236
33 WAP		*A. × cornutum* accessions
**Ability to flower**	**Percentage of flowering in 2018 ^3^**	**Accessions**
No or very rare flowering	10% or less	IPT215, IPT022, IPT216, IPT239, IPT240
Rare flowering	15–30%	IPT021, IPT212, IPT231, IPT237
Most accessions flower	40–60%	IPT211, IPT213, IPT217, IPT218, IPT225, IPT228, IPT229, IPT235, IPT238, IPT241
Obligatory flowering	70–100%	*A. × proliferum* accessions, IPT214, IPT176, IPT208, IPT226, IPT227, IPT230, from IPT232 to IPT234, IPT236, from IPT242 to IPT245

^1^ WAP–weeks after planting shallot accessions to flowering. ^2^ Accession code from ex situ collection (Institute of Agriculture and Tourism, IPTPO); ^3^ Percentage of plants that flowered in 2018 from 40 bulbs planted per accession.

**Table 2:** Qualitative generative shallot morphology descriptors for 32 flowering accessions.

Accession	Species	^1^ Flower Number in Inflorescence (FQL 1) ^3^	Anther Color (FQL 2)	Stamen Type ^4^ (FQL 3)	Pistil Type ^5^ (FQL 4)	Perianth Type ^6^ (FQL 5)	Inflorescence Type ^7^ (FQL 6)	Scape Type ^8^ (FQL 7)
IPT023	*A. × proliferum*	Few < 30 {1}	Green {5} ^2^	{1}	{2}	{2}	{2}	{2}
IPT210		Few < 30 {1}	Green {5}	{1}	{2}	{2}	{2}	{2}
IPT021	*A. × cornutum*	Many > 30 {2}	Yellow {1}	{3}	{2}	{3}	{1}	{1}
IPT211		Many > 30 {2}	Yellow {1}	{3}	{2}	{3}	{3}	{1}
IPT212		Many > 30 {2}	Yellow {1}	{3}	{2}	{3}	{3}	{1}
IPT213		Many > 30 {2}	Yellow {1}	{3}	{2}	{3}	{3}	{1}
IPT214		Many > 30 {2}	Yellow {1}	{3}	{2}	{3}	{3}	{1}
IPT176	*A. cepa* Aggregatum group	Many > 30 {2}	Green {5}	{2}	{1}	{1}	{1}	{1}
IPT208		Many > 30 {2}	Green {5}	{1}	{1}	{1}	{1}	{1}
IPT217		Many > 30 {2}	Green {5}	{1}	{1}	{1}	{1}	{1}
IPT218		Many > 30 {2}	Green {5}	{1}	{1}	{1}	{1}	{1}
IPT225		Many > 30 {2}	Green {5}	{2}	{1}	{1}	{1}	{1}
IPT226		Many > 30 {2}	Green {5}	{2}	{1}	{1}	{1}	{1}
IPT227		Many > 30 {2}	Green {5}	{2}	{1}	{1}	{1}	{1}
IPT228		Many > 30 {2}	Green {5}	{2}	{1}	{1}	{1}	{1}
IPT229		Many > 30{2}	Green {5}	{2}	{1}	{1}	{1}	{1}
IPT230	*A. cepa* Aggregatum group	Many > 30 {2}	Green {5} ^2^	{1}	{1}	{1}	{1}	{1}
IPT231		Many > 30 {2}	Green {5}	{2}	{1}	{1}	{1}	{1}
IPT232		Many > 30 {2}	Green {5}	{2}	{1}	{1}	{1}	{1}
IPT233		Many > 30 {2}	Green {5}	{2}	{1}	{1}	{1}	{1}
IPT234		Many > 30 {2}	Green {5}	{2}	{1}	{1}	{1}	{1}
IPT235		Many > 30 {2}	Green {5}	{1}	{1}	{1}	{1}	{1}
IPT236		Many > 30 {2}	Green {5}	{2}	{1}	{1}	{1}	{1}
IPT237		Many > 30 {2}	Green {5}	{2}	{1}	{1}	{1}	{1}
IPT238		Many > 30 {2}	Green {5}	{2}	{1}	{1}	{1}	{1}
IPT239		Many > 30 {2}	Green {5}	{2}	{1}	{1}	{1}	{1}
IPT240		Many > 30 {2}	Green {5}	{2}	{2}	{1}	{1}	{1}
IPT241		Many > 30 {2}	Green {5}	{2}	{2}	{1}	{1}	{1}
IPT242		Many > 30 {2}	Green {5}	{2}	{2}	{1}	{1}	{1}
IPT243		Many > 30 {2}	Green {5}	{1}	{1}	{1}	{1}	{1}
IPT244		Many > 30 {2}	Green {5}	{1}	{1}	{1}	{1}	{1}
IPT245		Many > 30 {2}	Green {5}	{1}	{1}	{1}	{1}	{1}

^1^ Descriptors in the table represent median value of observed flowering accessions (*n* = 5) according to ECPGR descriptors for *Allium* spp. and based on descriptors given in Puizina (2013); ^2^ Numbers in the curly brackets are identifiers for categorical variable responses given in ECPGR or in Puizina (2013). ^3^ Flower number in inflorescence: Few < 30 {1}, Many > 30 {2}. ^4^ Stamen type: Green, A. cepa type {1}; Green, simple {2}; Yellow, A. cepa type {3}. ^5^ Pistil type: Lower than stamens {1}; Taller than stamens {2}. ^6^ Perianth type: Starlike, green stripe {1}; Campanulate, green stripe {2}; Starlike, purple stripe {3}. ^7^ Inflorescence type: Round, no bulbils {1}; Prismatic, carrying bulbils {2}; Round, carrying bulbils {3}. ^8^ Scape type: Conic, hollow, simple {1}; Conic, hollow, carrying bulbils in several levels {2}.

**Table 3:** Quantitative flower morphology traits.

Species	Inflorescence Diameter (mm) (FQN1)	Flower Pedicle Length (mm) (FQN 2)	Stamen Length (mm) (FQN 3)	Petal Length (mm) (FQN 4)	Petal Diameter (mm) (FQN 5)	Scape Diameter (mm) (FQN6)
*A. × proliferum*	30.69 ^1^ ± 3.39 b ^2^	11.62 ± 1.37 ab	5.80 ± 0.24	4.21 ± 0.15 a	2.78 ± 0.11 a	24.87 ± 1.21 a
*A. cepa* Aggregatum group	42.83 ± 0.69 a	15.46 ± 0.39 a	5.93 ± 0.07	3.58 ± 0.04 b	2.12 ± 0.03 b	14.87 ± 0.3 b
*A. × cornutum*	38.64 ± 2.15 ab	10.15 ± 0.87 b	5.46 ± 0.15	4.10 ± 0.10 a	3.04 ± 0.07 a	9.69 ± 0.76 c
*p*-value ^3^	0.001	<0.001	ns	<0.001	<0.01	<0.01

^1^ Data represent mean ± SD for all accessions within one species. ^2^ The different letters within column denote significant difference by Tukey’s Unequal N HSD test, *p* < 0.05. ^3^ Analyses of variance between proposed species; ns—nonsignificant or significant at reported *p* value.

**Table 4:** Qualitative and quantitative bulb morphology descriptors for all 35 shallot accessions.

Accession	Species	^1^ Bulb Shape (BQL 1)	Bulblets (BQL 3)	Bulbs Per Cluster (BQL 2)	Bulb Skin Color (BQL 4)	Bulb Skin Thickness (BQL 5)	Bulb Flesh Color (BQL 6)	Bulbs in Cluster (BQN 1)	Cluster Weight (g) (BQN 2)	Bulb Diameter (mm) (BQN 3)
IPT023	*A. × proliferum*	Ovate {7} ^2^	Present {1}	Scarce {1}	Brown {5}	Medium	Violet/white {4}	2.00 ± 0.67	150.69 ± 32.78	42.10 ± 3.12
IPT210		Broad oval {4}	Absent {0}	Scarce {1}	Brown {5}	Medium	Violet/white {4}	2.20 ± 0.84	82.70 ± 26.69	55.55 ± 8.36
IPT021	*A. × cornutum*	Ovate {7}	Absent {0}	Medium {5}	Light brown {4}	Thick	Green/white {3}	14.89 ± 10.46	284.92 ± 95.53	44.19 ± 4.97
IPT022		Ovate {7}	Absent {0}	Few {3}	Light brown {4}	Thick	Green/white {3}	5.90 ± 2.51	149.50 ± 37.26	43.03 ± 4.89
IPT211		Ovate {7}	Absent {0}	Medium {5}	Light brown {4}	Thick	Green/white {3}	11.40 ± 6.17	218.30 ± 36.96	42.37 ± 5.88
IPT212		Ovate {7}	Absent {0}	Few {3}	Light brown {4}	Thick	Green/white {3}	7.70 ± 3.59	185.40 ± 52.83	41.93 ± 5.17
IPT213		Ovate {7}	Absent {0}	Few {3}	Light brown {4}	Thick	Green/white {3}	5.20 ± 1.14	196.65 ± 15.92	47.83 ± 4.35
IPT214		Ovate {7}	Absent {0}	Few {3}	Light brown {4}	Thick	Green/white {3}	9.40 ± 4.97	208.70 ± 37.27	43.77 ± 4.01
IPT215		Ovate {7}	Absent {0}	Few {3}	Light brown {4}	Thick	Green/white {3}	7.20 ± 3.71	163.85 ± 31.50	42.4 ± 4.11
IPT176	*A. cepa* Aggregatum group	Broad oval {4}	Present {1}	Medium {5}	Light brown {4}	Thin	Violet/white {4}	8.70 ± 2.87	293.20 ± 66.66	57.55 ± 8.01
IPT208		Rhomboid {3}	Absent {0}	Medium {5}	Light brown {4}	Thin	Violet/white {4}	14.70 ± 5.96	277.80 ± 94.32	40.23 ± 7.13
IPT216		Broad oval {4}	Present {1}	Medium {5}	Light brown {4}	Thin	Violet/white {4}	15.00 ± 4.32	242.00 ± 56.85	44.41 ± 4.83
IPT217		Broad oval {4}	Present {1}	Medium {5}	Light violet {8}	Thin	Violet/white {4}	14.50 ± 8.09	352.55 ± 91.85	52.92 ± 9.69
IPT218		Broad oval {4}	Present {1}	Medium {5}	Light violet {8}	Thin	Violet/white {4}	11.90 ± 3.21	274.70 ± 53.94	53.29 ± 7.04
IPT225		Broad oval {4}	Present {1}	Medium {5}	Light violet {8}	Thin	Violet/white {4}	13.80 ± 5.90	327.06 ± 163.17	54.75 ± 11.43
IPT226		Broad oval {4}	Present {1}	Medium {5}	Light violet {8}	Thin	Violet/white {4}	15.50 ± 4.45	254.45 ± 75.00	42.41 ± 5.50
IPT227		Ovate {7}	Present {1}	Scarce {1}	Yellow {2}	Thin	White {1}	5.10 ± 2.38	128.95 ± 34.12	42.57 ± 9.26
IPT228		Broad oval {4}	Present {1}	Medium {5}	Light violet {8}	Thin	Violet/white {4}	11.60 ± 3.10	267.50 ± 67.80	47.90 ± 5.52
IPT229		Broad oval {4}	Present {1}	Medium {5}	Light violet {8}	Thin	Violet/white {4}	8.78 ± 2.30	353.89 ± 94.77	55.23 ± 10.02

^1^ Descriptors in the table represent mean value ± SD of all observed accessions (*n* = 10) according to European Cooperative Programme for Plant Genetic Resources (ECPGR) descriptors for *Allium* spp. ^2^ Numbers in the curly brackets are identifiers for categorical variable responses given in ECPGR.

**Table 5:** Quantitative vegetative and bulb morphology traits.

Species	Leaf Length (cm) (VQN 1)	Leaf Diameter (mm) (VQN 2)	Bulbs in Cluster (BQN 1) ^1^	Cluster Weight (g) (BQN 2)	Bulb Diameter (mm) (BQN 3)
*A. × proliferum*	38.99 ^1^ ± 1.39 a ^2^	18.71 ± 0.64 a	2.01 ± 1.26 c	128.03 ± 22.22 c	51.06 ± 2.16 a
*A. cepa* Aggregatum PO ^3^	36.29 ± 0.37 ab	10.66 ± 0.17 b	12.10 ± 0.34 a	273.12 ± 5.94 a	48.63 ± 0.58 a
*A. cepa* Aggregatum SH	34.60 ± 0.76 b	9.24 ± 0.35 c	7.67 ± 0.69 b	171.62 ± 12.17 bc	40.19 ± 1.18 b
*A. × cornutum*	35.53 ± 0.64 ab	8.63 ± 0.30 c	8.81 ± 0.52 b	201.05 ± 10.28 b	43.65 ± 1.00 b
*p*-value ^4^	0.029	<0.01	<0.01	<0.01	<0.001

^1^ Data represent mean ± SD for accession (*n* = 10). ^2^ The different letters within column denote significant difference by Tukey’s Unequal N HSD test, *p* < 0.05. ^3^ *A. cepa* Aggregatum PO—potato onion type of bulb; SH—shallot type of bulb. ^4^ Analyses of variance between proposed species significant at reported *p* value.

## Data Availability

Data is contained within the article or supplementary materials. The data presented in this study are available in Supplementary Materials.

## Supplementary Materials

The following are available online at https://www.mdpi.com/2223-7747/10/1/60/s1, Table S1: Croatian shallot landraces collection sites, assigned with accession code from ex-situ collection (IPTPO) and Croatian Plant Genetic Resources Database code; Table S2: Quantitative generative shallot morphology descriptors for 32 flowering accessions; Table S3: PCA sum of variance and loadings for 32 flowering shallot accessions; Table S4: Qualitative and quantitative vegetative morphology descriptors for all 35 shallot accessions.
