# Supplementary material for: Shallot Species and Subtypes Discrimination Based on Morphology Descriptors
Source: Plants (Basel). 2020 Dec 29;10(1):60. doi: 10.3390/plants10010060 (PMC7823654; doi:10.3390/plants10010060)
Supplement: Supplementary file 1 [file plants-10-00060-s001.pdf]

Table S1 Croatian shallot landraces collection sites, assigned with accession code from *ex-situ* collection (IPTPO) and Croatian Plant Genetic Resources Database code

Accession	Species	Croatian Plant Genetic Resources Database	Year of conservation	Origin	Geographic coordinates	Flowering DAP[1] in 2018	Flowering WAP[1] in 2018	Accession flowering in 2018 (%)[3]
IPT023	*A. × proliferum*	VEG00107	2014	Katun Trviški	45°15'41.0"N 13°52'18.1"E	230	32	100
IPT210		VEG00113	2017	Zamask	45°19'25.4"N 13°52'36.8"E	230	32	80
IPT021	*A. × cornutum*	VEG00105	2014	Rijeka	45°20'48.7"N 14°24'26.1"E	235	33	15
IPT022		VEG00106	2014	Krmed	45°04'58.8"N 13°49'09.8"E	233	33	2,5
IPT211			2015	Zemunik	44°06'30.0"N 15°22'23.0"E	233	33	60
IPT212			2015	Opuzen	43°01'02.4"N 17°33'40.8"E	233	33	20
IPT213			2015	Podstrana	43°29'22.8"N 16°32'57.8"E	235	33	60
IPT214			2015	Imotski	43°26'46.4"N 17°12'50.6"E	233	33	70
IPT215			2015	Drašnice	43°13'09.3"N 17°06'33.3"E	nd2	nd2	nd
IPT176	*A. cepa* Aggregatum group		2016	Debelo brdo	44°39'33.6"N 15°41'17.1"E	221	31	80
IPT208		VEG00112	2015	Kaštelir	45°18'06.8"N 13°41'13.9"E	226	32	100
IPT216		VEG00114	2015	Nova Gradiška	45°15'21.0"N 17°22'53.9"E	238	33	2,5
IPT217		VEG00110	2015	Umag	45°26'12.5"N 13°31'33.2"E	230	32	60
IPT218		VEG00111	2015	Umag	45°26'12.5"N 13°31'33.2"E	228	32	40
IPT225			2017	Mače	46°05'43.0"N 16°02'38.0"E	226	32	60
IPT226			2017	Pregrada	46°09'54.8"N 15°45'04.0"E	221	31	70
IPT227			2017	Novi Golubovec	46°10'23.0"N 15°59'00.3"E	221	31	100
IPT228			2017	Zabok	46°01'20.9"N 15°54'05.9"E	221	31	40
IPT229			2017	Oroslavje	45°59'39.4"N 15°54'56.9"E	221	31	40
IPT230			2017	Desinić	46°08'54.8"N 15°40'09.1"E	226	32	100
IPT231			2017	Desinić	46°08'54.8"N 15°40'09.1"E	226	32	30
IPT232			2017	Oroslavje	45°59'39.4"N 15°54'56.9"E	226	32	70
IPT233			2017	Pregrada	46°09'54.8"N 15°45'04.0"E	226	32	70
IPT234			2017	Pregrada	46°09'54.8"N 15°45'04.0"E	226	32	90
IPT235			2017	Radoboj	46°09'54.9"N 15°55'05.5"E	226	32	40
IPT236			2017	Oroslavje	45°59'39.4"N 15°54'56.9"E	226	32	70
IPT237			2017	Donja Stubica	45°58'50.2"N 15°58'12.4"E	221	31	20
IPT238			2017	Donja Stubica	45°58'50.2"N 15°58'12.4"E	221	31	50
IPT239			2017	Oroslavje	45°59'39.4"N 15°54'56.9"E	221	31	10
IPT240			2017	Pregrada	46°09'54.8"N 15°45'04.0"E	221	31	10
IPT241			2017	Gornje Jesenje	46°12'20.3"N 15°54'31.0"E	221	31	60
IPT242			2017	Gornje Jesenje	46°12'20.3"N 15°54'31.0"E	221	31	90
IPT243			2017	Mihovljani	46°07'59.9"N 15°58'08.1"E	221	31	80
IPT244			2017	Novi Golubovec	46°10'23.0"N 15°59'00.3"E	221	31	90
IPT245			2017	Donja Stubica	45°58'50.2"N 15°58'12.4"E	221	31	100

[1] DAP/WAP– days/weeks after planting necesary to enter flowering period; [2] nd – no data on flowering recorded; [3] From 40 bulbs planted per accession, the percentage that produced flowers.

Table S2 Quantitative generative shallot morphology descriptors for 32 flowering accessions

Accession	Species	[1]Inflorescence diameter (FQN1)	Flower pedicle length (FQN 2)	Stamen length (FQN 3)	Petal length (FQN 4)	Petal diameter (FQN 5)	Scape diameter (FQN6)
IPT023	*A.* × *proliferum*	20.83 ± 4.75	9.23 ± 1.86	5.98 ± 0.84	4.41 ± 0.21	2.85 ± 0.37	21.40 ± 3.95
IPT210		40.54 ± 7.32	14.02 ± 3.73	5.63 ± 0.71	4.02 ± 0.58	2.71 ± 0.20	13.34 ± 1.13
IPT021	*A.* × *cornutum*	41.59 ± 3.68	11.74 ± 1.73	5.44 ± 0.44	4.26 ± 0.60	3.16 ± 0.27	9.12 ± 1.35
IPT211		38.91 ± 3.43	11.29 ± 1.63	5.61 ± 0.33	4.36 ± 0.22	3.13 ± 0.27	10.00 ± 2.31
IPT212		33.14 ± 3.90	7.76 ± 1.55	5.39 ± 0.60	4.18 ± 0.17	2.92 ± 0.27	9.60 ± 2.22
IPT213		36.35 ± 6.37	8.53 ± 1.78	5.43 ± 0.31	3.95 ± 0.50	2.87 ± 0.38	12.60 ± 2.32
IPT214		43.19 ± 3.71	11.45 ± 1.86	5.42 ± 0.25	3.77 ± 0.31	3.10 ± 0.22	9.80 ± 1.48
IPT176	*A. cepa* Aggregatum group	41.65 ± 3.69	14.20 ± 2.69	5.97 ± 0.61	3.31 ± 0.61	2.20 ± 0.35	16.50 ± 1.87
IPT208		53.48 ± 3.54	23.47 ± 4.17	6.74 ± 0.33	4.17 ± 0.17	2.14 ± 0.15	20.38 ± 3.40
IPT217		46.23 ± 10.63	19.04 ± 3.23	6.68 ± 1.08	4.15 ± 0.74	1.71 ± 0.44	13.28 ± 3.74
IPT218		48.20 ± 3.64	15.14 ± 1.76	6.31 ± 0.98	3.64 ± 0.57	2.23 ± 0.18	10.96 ± 2.49
IPT225		39.00 ± 4.04	14.89 ± 0.57	6.18 ± 0.75	3.12 ± 0.25	2.26 ± 0.35	15.40 ± 3.31
IPT226		43.82 ± 7.68	15.25 ± 0.97	5.93 ± 0.66	3.83 ± 0.51	2.17 ± 0.27	12.10 ± 1.91
IPT227		59.40 ± 5.60	21.61 ± 1.26	5.93 ± 0.55	3.64 ± 0.47	1.92 ± 0.2	20.40 ± 2.67
IPT228		59.43 ± 6.76	21.30 ± 3.44	6.12 ± 0.44	3.50 ± 0.36	1.98 ± 0.21	12.50 ± 2.42
IPT229		49.64 ± 10.10	15.29 ± 5.10	5.92 ± 1.22	3.79 ± 0.46	2.26 ± 0.18	15.40 ± 4.14
IPT230		66.14 ± 4.26	20.39 ± 2.40	6.87 ± 0.46	4.34 ± 0.23	2.17 ± 0.11	15.50 ± 1.84
IPT231		36.97 ± 8.00	14.89 ± 2.20	5.62 ± 0.34	3.68 ± 0.29	2.50 ± 0.37	13.40 ± 2.59
IPT232		41.01 ± 5.90	13.98 ± 1.29	6.06 ± 0.50	3.50 ± 0.28	2.36 ± 0.60	12.55 ± 1.64
IPT233		35.11 ± 6.47	12.86 ± 3.28	6.24 ± 0.75	3.06 ± 0.29	2.12 ± 0.37	15.10 ± 1.97
IPT234		41.90 ± 7.24	14.62 ± 3.02	5.50 ± 0.82	3.38 ± 0.40	2.15 ± 0.39	14.40 ± 2.22
IPT235		36.48 ± 4.78	10.65 ± 3.39	5.14 ± 1.20	3.62 ± 0.37	2.26 ± 0.46	15.00 ± 2.67
IPT236		42.43 ± 1.78	14.89 ± 1.05	6.17 ± 0.45	3.76 ± 0.45	2.41 ± 0.14	15.10 ± 2.77
IPT237		32.44 ± 2.97	13.71 ± 4.07	6.23 ± 1.00	3.58 ± 0.40	2.23 ± 0.65	11.70 ± 2.31
IPT238		30.96 ± 3.92	12.92 ± 4.35	5.80 ± 1.04	3.41 ± 0.28	2.26 ± 0.57	14.20 ± 2.04
IPT239		36.84 ± 6.41	12.99 ± 2.73	5.79 ± 0.55	3.40 ± 0.38	2.27 ± 0.20	14.20 ± 2.49
IPT240		27.97 ± 5.15	9.62 ± 1.43	5.71 ± 1.03	3.60 ± 0.40	1.92 ± 0.32	13.10 ± 2.96
IPT241		29.58 ± 5.08	10.05 ± 1.57	5.98 ± 0.64	3.65 ± 0.40	1.92 ± 0.29	15.50 ± 3.14
IPT242		28.12 ± 5.30	9.68 ± 1.10	5.84 ± 0.49	3.70 ± 0.29	1.88 ± 0.26	13.30 ± 2.00
IPT243		48.52 ± 7.23	18.51 ± 4.42	5.14 ± 0.67	3.08 ± 0.46	1.75 ± 0.17	13.60 ± 2.72
IPT244		48.5 ± 9.56	17.72 ± 3.91	5.26 ± 0.48	3.22 ± 0.47	2.05 ± 0.15	15.30 ± 3.53
IPT245		47.04 ± 7.52	18.79 ± 5.80	5.08 ± 0.54	3.41 ± 0.34	2.02 ± 0.19	16.30 ± 3.33

[1]Descriptors in the table represent mean ± SD value of observed flowering accessions (n=5) according to ECPGR descriptors for *Allium* spp. and based on descriptors proposed by Puizina (2013).

Table S3 PCA sum of variance and loadings for 32 flowering shallot accessions

	PC1 [2]	***PC2***	***PC3***	***PC4***
Eigenvalues[1]	8.8954	5.8912	2.4543	1.2144
% Total variance	40.4334	26.7782	11.156	5.5202
Cumulative eigenvalue	8,8954	14.7866	17.2409	18.4553
Cumulative %	40.4334	67.2117	78.3676	83.8878
FQL1[3]	0.1148[4]	**-0.3503**	-0.0347	-0.0188
FQN1	0.1157	-0.0829	**0.4365**	0.2443
FQN2	0.1821	0.0056	**0.4081**	0.1963
FQN3	0.0761	0.0347	0.2532	**0.5800**
FQN4	-0.1498	0.0418	0.2477	**0.3173**
FQN5	**-0.2416**	-0.0121	0.1067	0.2064
FQN6	0.0474	**0.2932**	0.0333	-0.0618
FQL2 {1}[5]	**-0.2862**	-0.2001	0.0591	0.0063
FQL2 {5}	**0.2862**	0.2001	-0.0591	-0.0063
FQL3 {3}	**-0.2862**	-0.2001	0.0591	0.0063
FQL3 {1}	0.0846	0.0892	**0.4648**	**-0.4753**
FQL3 {2}	0.1302	0.0633	**-0.4727**	**0.4348**
FQL4 {2}	**-0.2871**	0.0424	-0.1357	0.0287
FQL4 {1}	**0.2871**	-0.0424	0.1357	-0.0287
FQL5 {3}	**-0.2862**	-0.2001	0.0591	0.0063
FQL5 {2}	-0.1285	**0.3758**	0.0281	0.0092
FQL5 {1}	**0.3266**	-0.0443	-0.0683	-0.0110
FQL6 {1}	**0.3038**	-0.0814	-0.0578	0.0133
FQL6 {2}	-0.1285	**0.3758**	0.0281	0.0092
FQL6 {3}	**-0.2645**	-0.1789	0.0476	-0.0224
FQL7 {1}	0.1285	**-0.3758**	-0.0281	-0.0092
FQL7 {2}	-0.1285	**0.3758**	0.0281	0.0092

[1] Principal Components Analysis sum of variance.
[2] Principal Component significant if eigenvalue > 1.
[3] Variable/descriptor according to ECPGR descriptors for *Allium* spp. and based on descriptors proposed by Puizina (2013).
[4] Eigenvectors obtained in the PCA analyses; significant correlations in bold.
[5] Number in the curly brackets represents value of the expression of the ordinal and categorical variables given in ECPGR or in Puizina (2013).

Table S4 Qualitative and quantitative vegetative morphology descriptors for all 35 shallot accessions

Accession	Species	[1]Foliage color (VQL 1)	Foliage attitude (VQL 2)	Foliage cracking (VQL 3)	Degree of leaf waxiness (VQL 4)	Leaf length (cm) (VQN 1)	Leaf diameter (mm) (VQN 2)
IPT023	*A.* × *proliferum*	Dark green {5}[2]	Erect {7}	Weak {3}	Strong {7}	45.00 ± 7.71	21.40 ± 3.95
IPT210		Dark green {5}	Erect {7}	Weak {3}	Strong {7}	26.98 ± 1.42	13.34 ± 1.13
IPT021	*A.* × *cornutum*	Light green {1}	Prostrate {3}	Weak {3}	Medium {5}	32.90 ± 3.98	8.10 ± 1.73
IPT022		Light green {1}	Prostrate {3}	Weak {3}	Medium {5}	33.45 ± 1.52	7.50 ± 1. 35
IPT211		Green {3}	Intermediate {5}	Weak {3}	Strong {7}	36.55 ± 2.89	9.90 ± 2.51
IPT212		Green {3}	Intermediate {5}	Weak {3}	Medium {5}	36.40 ± 1.26	8.70 ± 1.34
IPT213		Grey-green {4}	Intermediate {5}	Weak {3}	Medium {5}	38.45 ± 2.49	10.60 ± 1.43
IPT214		Green {3}	Prostrate {3}	Weak {3}	Medium {5}	35.35 ± 2.53	7.90 ± 0.88
IPT215		Green {3}	Prostrate {3}	Weak {3}	Week {3}	35.60 ± 2.23	7.70 ± 1.34
IPT176	A. cepa Aggregatum group	Bluish green {6}	Intermediate {5}	Weak {3}	Strong {7}	38.35 ± 3.91	12.80 ± 1.48
IPT208		Green {3}	Erect {7}	Strong {7}	Strong {7}	38.53 ± 5.13	13.20 ± 2.74
IPT216		Green {3}	Intermediate {5}	Medium {5}	Week {3}	36.85 ± 4.02	10.50 ± 2.01
IPT217		Grey-green {4}	Intermediate {5}	Medium {5}	Strong {7}	42.10 ± 6.48	10.20 ± 2.62
IPT218		Grey-green {4}	Prostrate {3}	Weak {3}	Strong {7}	37.70 ± 5.77	9.10 ± 1.91
IPT225		Bluish green {6}	Prostrate {3}	Weak {3}	Strong {7}	37.15 ± 4.38	11.30 ± 2.54
IPT226		Green {3}	Prostrate {3}	Strong {7}	Week {3}	35.60 ± 6.09	10.60 ± 1.65
IPT227		Green {3}	Prostrate {3}	Strong {7}	Week {3}	34.25 ± 5.12	12.10 ± 2.56
IPT228		Bluish green {6}	Intermediate {5}	Strong {7}	Week {3}	36.05 ± 5.38	11.20 ± 1.99
IPT229		Bluish green {6}	Prostrate {3}	Weak {3}	Medium {5}	37.50 ± 4.24	10.90 ± 2.02
IPT230		Green {3}	Prostrate {3}	Weak {3}	Medium {5}	39.05 ± 5.34	12.00 ± 1.83
IPT231		Dark green {5}	Intermediate {5}	Medium {5}	Week {3}	35.00 ± 4.47	11.50 ± 1.51
IPT232		Dark green {5}	Intermediate {5}	Medium {5}	Week {3}	34.95 ± 3.72	11.65 ± 1.53
IPT233		Dark green {5}	Prostrate {3}	Medium {5}	Medium {5}	37.05 ± 4.72	10.80 ± 2.15
IPT234		Bluish green {6}	Intermediate {5}	Medium {5}	Medium {5}	39.15 ± 7.33	11.80 ± 3.36
IPT235		Bluish green {6}	Intermediate {5}	Weak {3}	Strong {7}	39.30 ± 3.86	11.10 ± 2.02
IPT236		Green {3}	Intermediate {5}	Weak {3}	Strong {7}	34.55 ± 5.57	9.40 ± 2.50
IPT237		Green {3}	Prostrate {3}	Medium {5}	Medium {5}	28.85 ± 7.65	8.00 ± 1.56
IPT238		Bluish green {6}	Prostrate {3}	Medium {5}	Medium {5}	33.60 ± 2.85	9.30 ± 1.64
IPT239		Green {3}	Prostrate {3}	Medium {5}	Week {3}	33.15 ± 2.31	6.80 ± 1.14
IPT240		Green {3}	Prostrate {3}	Medium {5}	Medium {5}	34.35 ± 3.42	8.50 ± 1.84
IPT241		Bluish green {6}	Intermediate {5}	Weak {3}	Medium {5}	35.30 ± 3.31	9.20 ± 1.99
IPT242		Green {3}	Prostrate {3}	Medium {5}	Week {3}	33.15 ± 2.42	9.30 ± 1.77
IPT243		Bluish green {6}	Prostrate {3}	Medium {5}	Medium {5}	34.85 ± 6.31	9.80 ± 1.81
IPT244		Dark green {5}	Intermediate {5}	Medium {5}	Medium {5}	32.70 ± 3.51	9.50 ± 1.78
IPT245		Bluish green {6}	Prostrate {3}	Medium {5}	Medium {5}	35.95 ± 7.57	9.60 ± 2.32

[1]Descriptors in the table represent mean (quantitative) ± SD and median (qualitative) value of all observed accessions (n=10) according to ECPGR descriptors for *Allium* spp. [2] Number in the curly brackets represents value of the expression of the ordinal and categorical variables given in ECPGR or in Puizina (2013).
